# Behavioural Impact of Parental Presence Versus Absence in Paediatric Dentistry: A Systematic Review

**DOI:** 10.3390/dj14010033

**Published:** 2026-01-05

**Authors:** M. Angeles Vello-Ribes, J. Ignacio Aura-Tormos, Carolina Valero-Contelles, M. Dolores Casaña-Ruiz, Montserrat Catala-Pizarro

**Affiliations:** Department of Dentistry, Faculty of Medicine and Dentistry, University of Valencia, 46010 Valencia, Spain; m.angeles.vello@uv.es (M.A.V.-R.); maria.d.casana@uv.es (M.D.C.-R.); montserrat.catala@uv.es (M.C.-P.)

**Keywords:** parental presence, paediatric dentistry, child behaviour, dental anxiety, behaviour guided technique, parenting style, non-pharmacological techniques

## Abstract

**Background/Objectives**: Parental presence or absence (PPA) in the dental operatory remains a central issue in paediatric behaviour guidance, commonly employed as a non-pharmacological approach, yet frequently perceived as a professional dilemma among paediatric dentists. Its behavioural impact on children during dental treatment remains debated. This systematic review evaluates the influence of PPA on children’s behaviour in dental settings and explores moderating factors. **Methods**: A PRISMA-guided systematic review was conducted in PubMed, Web of Science, and Scopus for primary studies published between 2005 and 2025. Eligibility criteria included clinical studies involving paediatric patients primarily aged 2–14 years, comparing parental presence vs. absence during dental visits. **Results**: The 16 included studies consisted of randomized controlled trials (*n* = 9), cohort studies (*n* = 3), and analytical cross-sectional designs (*n* = 4). Findings were heterogeneous; nine of sixteen studies reported that PPA improved cooperative behaviour, particularly in younger children (ages 4–6), those with higher IQ, or those with initially negative behaviour. Five studies found no significant effect, while two noted increased anxiety or disruptive behaviour with parental presence. Parenting style and cultural context influenced outcomes, with authoritative styles associated with better cooperation. **Conclusions**: PPA can enhance behaviour in specific subgroups but lacks universal benefits. Paediatric dentists should individualize its use according to each child’s developmental stage, emotional profile, and family dynamics, particularly parenting style, to optimize outcomes.

## 1. Introduction

Managing paediatric patient behaviour is crucial in paediatric dentistry, as treatment success depends largely on child cooperation [1]. Among non-pharmacological behaviour guidance techniques, parental presence versus absence (PPA) plays a potentially key role in shaping behaviour during dental visits [1,2]. Rapport must be built from the child’s entry into the clinic, and the parent’s role has gained relevance amid debate over whether their presence supports or hinders cooperation [3]. Although evidence is limited, findings diverge: some studies report that PPA calms young children, while others highlight that parental anxiety or over-involvement can worsen compliance [2,4]. Consequently, dentists must evaluate both child and parent-related factors [5].

Child behaviour in the dental setting is influenced by multiple variables, including parental attitudes, prior dental experiences, separation from caregivers, cognitive level, age, gender, cultural background, visit duration, and timing [4]. Anxiety may stem from negative parental messages or using dental treatment as punishment [2,3,4,5]. Around age three, separation anxiety often begins, making parental presence helpful for younger children. In contrast, older children may be negatively affected by visibly anxious parents, as their greater cognitive maturity and emotional attunement make them more likely to internalise parental stress and interpret the situation as threatening [2,4,5].

Despite these multiple behavioural influences, a central and long-standing question in paediatric dentistry remains whether parental presence or absence optimally supports children’s cooperation and emotional regulation during dental treatment.

Although variables such as Intelligence Quotient (IQ), age, and gender have been examined, findings remain inconclusive. Western studies report minimal gender differences, while cultural and ethnic background significantly shapes parent–child–dentist interactions [6]. Other contextual factors such as attention span and appointment timing also matter—morning visits tend to be more effective with younger patients [7]. Beyond these influences, variables, non-cooperative behaviour may arise from deeper emotional causes, including chronic illness, past trauma, introversion, specific phobias, or undiagnosed psychological conditions. These challenges are exacerbated when parents normalize disruptive behaviour or reject psychological explanations [2,3,4,5,6,7]. Dentists must also remain alert to signs of abuse or neglect, in line with their legal and ethical responsibilities [4,5,6,7].

The way families perceive and accept PPA is also shaped by sociocultural context. Cultural norms influence parental expectations regarding medical care [6]. In Western societies, trends toward overprotection may restrict children’s autonomy and foster unrealistic behavioural expectations [7], particularly, among first-time mothers [8]. Many parents prefer to accompany their child during dental procedures—especially when they perceive that separation might trigger anxiety in either the child or themselves, yet this presence is not always beneficial. Parental fear or controlling behaviour can elicit negative reactions from the child [3]. Nevertheless, their desire to remain often reflects protective instincts rather than distrust [9]. Recognizing these emotional dynamics is crucial, and paediatric dentists should prioritize empathetic, transparent communication to build trust with both child and parents [7,8,9].

In recent decades, legal and healthcare reforms have strengthened parents’ rights to accompany their children, promoting a more child-centred model of care [10]. Although this approach is widely adopted in medicine, paediatric dentistry continues to rely on traditional behaviour guidance techniques that may not fully meet current parental expectations [2]. Increased demand for transparency and involvement—driven partly by work-related time constraints—has made parents more informed and assertive [9,10,11,12]. Consequently, dental professionals must reconsider how PPA affects paediatric behaviour and adapt their management strategies accordingly [11,12,13,14,15,16].

Positive involvement supports cooperative behaviours, whereas parental stress, negative experiences, and depressive symptoms can heighten a child’s anxiety and emotional vulnerability [17,18]. In this context, open communication regarding treatment plans and behavioural expectations is essential, although excessive parental involvement should be avoided, as it can disrupt management [2,19,20,21,22]. A thorough understanding of the child’s temperament, previous medical experiences, and cognitive-emotional development enables tailored approaches [9,23].

Traditional techniques such as hand-over-mouth or voice control have declined due to shifting societal norms [12,24], increasing the relevance of PPA. Recent studies support its effectiveness: parental presence improves cooperation in children aged 4–6 years with disruptive behaviour [25,26,27] and in those with higher cognitive ability [28]. ALDhelai et al. [28] further compared active versus passive parental presence, finding better outcomes when parents adopted an active role, highlighting that the quality of involvement may be as important as its mere presence or absence. Additional benefits have been reported during specific procedures, such as local anaesthesia [29], with structured formats like the Parent Present/Absent Technique proving effective in children aged 6–9 [29]. Overall, greater parental engagement tends to enhance cooperation [30], and even when anxiety reduction is modest, many children report feeling emotionally safer with a parent present [31].

Although recent findings are encouraging, inconsistencies remain. Further research is required to clarify how factors such as age and cognitive development influence the effectiveness of PPA. In our community, healthcare regulations have enshrined the legal right of patients to be accompanied during clinical procedures—whenever feasible—as a core element of patient-centred care [32].

In light of these gaps and the need for consistent, evidence-based guidance, this systematic review aims to determine whether parental presence during paediatric dental treatment influences children’s behavioural and anxiety-related responses, compared with treatment delivered without parental presence, among patients aged 2–14 years assessed through validated behavioural measures.

## 2. Materials and Methods

A systematic review was conducted to analyse the influence of parental presence on children’s behaviour during dental visits. The review followed the 2020 PRISMA guidelines (Preferred Reporting Items for Systematic Reviews and Meta-Analyses), with the corresponding checklist provided as Supplementary Material (Table S1) [33]; and employed the Joanna Briggs Institute (JBI) Critical Appraisal Tools for quality assessment [34]. The protocol was registered in the International Prospective Register of Systematic Reviews (PROSPERO) under the registration number CRD420250652167.

**Eligibility Criteria**. Studies were eligible if they involved paediatric patients primarily aged 2 to 14 years, reflecting the typical age range managed in dental settings where behaviour guidance is critical. Additional inclusion criteria required assessment of the influence of PPA on children’s behaviour in a clinical dental context, and the study had to constitute primary research, specifically randomised controlled trials (RCTs), cohort studies, or analytical cross-sectional designs. Only original articles with full-text availability were selected. Exclusion criteria encompassed secondary sources such as systematic reviews, meta-analyses, books, and conference abstracts. No date limits were applied, and the search was restricted to studies published in English.

**Data Sources**. A comprehensive search was carried out in the following electronic databases: PubMed, Web of Science, and Scopus. Inclusion and exclusion criteria were applied during the screening process.

**Search Strategy**. The search strategy was developed based on the PICO framework, targeting children undergoing dental treatment (P), with parental presence during the appointment as the intervention (I), various formats of parental presence vs. its absence as the comparator (C), and behavioural outcomes as the focus (O). Boolean operators and truncation were used to combine keywords across three databases: PubMed, Web of Science, and Scopus. The search included the following terms: [child OR infant OR youth OR kid], [parental presence OR parental absence OR parental involvement OR parental participation OR parental separation OR non-pharmacological behavior guidance techniques OR non pharmacological behavior guidance techniques OR BGT OR non-pharmacological management OR non pharmacological management], [behavior OR behaviour OR fear OR phobia OR anxiety OR odontophobia]*, and [dental OR pediatric dentistry OR paediatric dentistry]. Search syntax was adapted to each platform [e.g., MeSH in PubMed, TS = in Web of Science, and TITLE-ABS-KEY in Scopus].

**Data Extraction**. Two reviewers (C.V. and MA.V.) independently extracted information on study characteristics, outcomes, and potential moderators using a piloted form; Any discrepancies were resolved through discussion and consensus, with a third author (J.I.A.T.) consulted when necessary. Although both title/abstract screening and full-text assessment were performed independently by two reviewers, inter-reviewer agreement statistics were not calculated due to the qualitative and iterative nature of the screening process.

**Data Synthesis**. A structured narrative synthesis was undertaken following PRISMA guidance. Studies were systematically grouped according to (1) age-related behavioural outcomes, (2) comparisons between parental presence versus absence formats, (3) study design (RCT, cohort, cross-sectional), and (4) moderating variables such as parenting style, anxiety level, or cognitive ability. This analytic approach enabled identification of patterns and divergences across heterogeneous methodologies.

Moreover, quantitative pooling was not performed because the included studies displayed substantial methodological and clinical heterogeneity, including wide variability in behavioural outcome measures, inconsistent reporting formats, heterogeneous age ranges, differing parental involvement protocols (active, passive, alternating), and diverse study designs. In addition, several studies reported outcomes in non-standardised or purely descriptive formats, which prevented the derivation of any common metric. These differences precluded the calculation of comparable effect sizes and made any form of quantitative aggregation statistically inappropriate and potentially misleading.

**Quality Assessment**. The methodological quality and risk of bias of the included studies were evaluated using JBI Critical Appraisal Tools [34], with the appropriate checklist applied according to each study design. Randomized controlled trials, cohort studies, and cross-sectional studies were assessed using their corresponding JBI tools to ensure consistency and rigor. To allow standardised interpretation across designs, studies were categorised according to the proportion of appraisal items rated “Yes,” using pre-specified thresholds: ≥80% “Yes” responses were classified as high quality, 50–79% as moderate quality, and <50% as low quality. These thresholds were defined a priori and did not influence study eligibility.

## 3. Results

### 3.1. Study Selection and Flow Diagram

The selection process followed the PRISMA guidelines. The initial search yielded a total of 333 records: 126 from PubMed, 83 from Web of Science, and 124 from Scopus. After removing 220 duplicates, 113 unique articles remained for screening.

**Figure 1:**
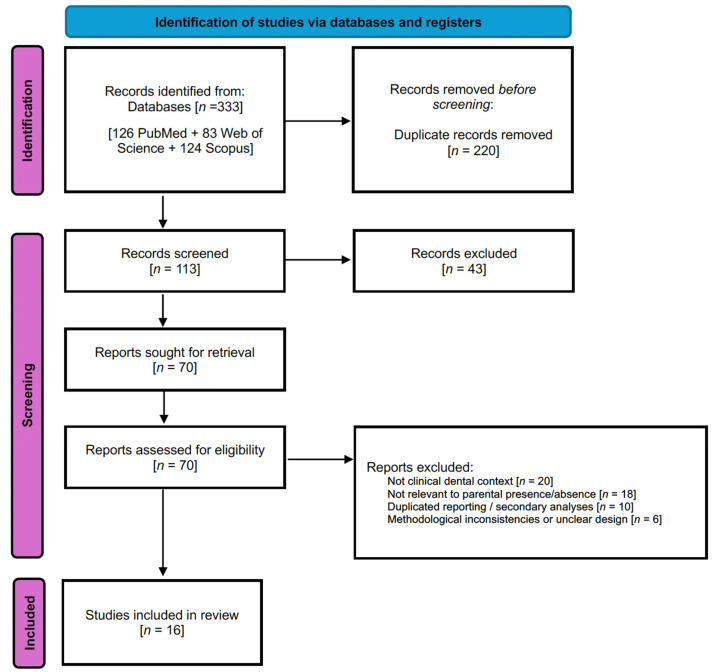
PRISMA Diagram flowchart.

Two independent reviewers screened titles and abstracts, excluding 43 records that did not meet the inclusion criteria. The remaining 70 articles underwent full-text evaluation. All included studies were appraised independently by both reviewers, with any disagreements resolved by discussion and consensus. Following full-text assessment 54 articles were excluded for the following reasons: non-clinical dental contexts (*n* = 20), lack of relevance to parental presence/absence (*n* = 18), duplicate or secondary analyses (*n* = 10), and significant methodological issues, such as absence of standardized behavioural measures, small or unrepresentative samples, or inadequate control of confounding factors (*n* = 6). As a result, 16 studies were included in the qualitative synthesis (Figure 1).

### 3.2. Qualitative Analysis

The 16 studies included in this systematic review were published between 2005 and 2025, and conducted across diverse cultural settings, including Greece, Iran, Bulgaria, the Netherlands, Egypt, Saudi Arabia, India, and Turkey. Study designs encompassed three cohort studies, nine randomized controlled trials, and four analytical cross-sectional studies.

Sample sizes ranged from 30 to 440 participants, with children aged 2–14 years. Behavioural outcomes were assessed in a variety of clinical contexts such as initial dental visits, preventive procedures, restorative treatments, pulpotomies, and extractions, both with and without local anaesthesia.

A broad range of validated behavioural and anxiety assessment tools was employed across studies, including the Frankl Behaviour Rating Scale (Frankl), Venham Anxiety Scale (VAS), Modified Dental Anxiety Scale (MDAS), Abeer Children Dental Anxiety Scale (ACDAS), Wong-Baker Faces Pain Rating Scale (WBFPS), Facial Image Scale (FIS), and other self-reported anxiety measures. Several studies also monitored physiological parameters such as heart rate (HR) and oxygen saturation (SatO_2_).

Behaviour management strategies varied considerably. Parental presence was most frequently applied in its active [PAP], passive [PPP], or alternating [PPAT] formats. Additional non-pharmacological techniques included Tell-Show-Do, positive reinforcement, voice control, modelling, and distraction (Table A1, Appendix A).

### 3.3. Quality Assessment of the Cohort Studies

The quality of the three cohort studies was assessed using the JBI checklist, which includes 11 items addressing key aspects of methodological rigor. As summarized in Table 1, all studies fulfilled most of the core criteria. Kotsanos et al. showed notable shortcomings in Q9–Q11, related to follow-up completeness and data analysis, resulting in an overall rating of moderate to low quality [19,20].

By contrast, Al-Namankany et al. achieved a higher score, with incomplete follow-up (Q10) as the only unmet criterion, and it was rated as moderate quality. This study demonstrated greater consistency in handling confounding variables (Q4–Q5), exposure measurement (Q6), and outcome assessment (Q8) [29]. Despite these limitations, the cohort evidence was considered methodologically adequate to contribute to conclusions about the behavioural impact of parental presence.

### 3.4. Quality Assessment of Randomized Controlled Trials

The randomized controlled trials were evaluated using the JBI checklist for RCTs, which comprises 13 indicators of methodological quality. As shown in Table 2, most studies achieved a moderate rating, providing a reasonable level of confidence in their findings.

Shindova et al. were rated as low quality due to unclear randomization procedures (Q1–Q4) and insufficiently described statistical analysis (Q13). Similarly, Ahuja et al. [3] received an uncertain rating because essential methodological details such as allocation concealment, blinding, and follow-up were not adequately reported [23]. The remaining RCTs presented only minor shortcomings, mainly related to blinding (Q2, Q6), but met most appraisal criteria. Overall, despite some methodological constraints the RCTs provided clinically relevant evidence on the behavioural effects of parental presence.

Additionally, some studies broadened the focus beyond the strict presence-absence dichotomy. For instance, Bagavathy et al. [30] explored “parental involvement” and its association with child compliance; however, the criteria used to classify different levels of involvement were not specified, making the results difficult to interpret objectively. While this study does not directly address PPA, it nonetheless suggests that parental participation, regardless of how it is defined, may influence children’s behavioural responses during dental treatment.

### 3.5. Quality Assessment of Cross-Sectional Studies

The four analytical cross-sectional studies were assessed using the JBI checklist, which includes 8 items. All four studies, Acharya et al. [4], Shiraz et al. [27], Bagavathy et al. [30], and Gera et al. [35], were rated as having moderate overall quality (Table 3).

They demonstrated consistent strengths in methodological clarity (Q1–Q4), valid outcome measurement (Q7), and appropriate statistical analysis (Q8). The most frequent limitation concerned confounding factors, as Q5 and Q6 were often unmet. Nonetheless, the cross-sectional studies provided valuable insights into variable relationships at specific time points and contributed meaningfully to the overall interpretation of evidence regarding parental presence in paediatric dental care.

## 4. Discussion

This review synthesised evidence from nine randomised controlled trials, three cohort studies, and four analytical cross-sectional studies, revealing notable variability in the behavioural effects of PPA during paediatric dental treatment. While a consistent advantage of PPA cannot be established across all contexts, several studies described improvements in specific subgroups or settings. This variability suggests that the effect of parental presence depends on a complex interaction of child, parent, and procedural factors, rather than operating as a uniform influence.

Overall, most included studies were rated as moderate quality according to the JBI criteria (68%), with only a minority classified as low or moderate–low quality (18%). This distribution indicates that, although the evidence base is acceptable, methodological limitations should be considered when interpreting these findings.

### 4.1. Behavioural Improvement in Younger Versus Older Children

Behavioural improvement in younger versus older children. Of the 16 included studies, nine reported statistically significant improvements in children’s behaviour associated with parental presence, particularly among younger children (3–6 years) or those who were initially uncooperative (Frankl scores 1–2) [3,4,19,20,27,28,30].

Kotsanos et al. [19,20] reported that parental presence improved behaviour mainly in younger children and in those with mild to moderate behavioural difficulties, whereas effects were limited in more severe cases. Consistent with this pattern, Acharya et al. [4] and Shiraz et al. [27] also observed better cooperation among children in the 3–6-year age range when a parent was present.

In contrast, no statistically significant improvement was observed among older children [21,22,23,25,26,31], suggesting that developmental maturity moderates the PPA effect.

Aldhelai et al. [28] also reported statistically significant improvement (*p* < 0.05) among preschoolers with higher cognitive ability, indicating that cognitive control facilitates the positive impact of parental presence.

### 4.2. Studies Reporting No Significant Differences

In contrast, several studies—including Afshar et al. [22], Shindova and Belcheva [23], Boka et al. [25,26], and Karaca and Şirinoğlu Capan [31]—reported no statistically significant differences in children’s behaviour or anxiety between parental presence and absence.

No measurable differences in anxiety or cooperation were observed across either non-invasive (prophylaxis) or invasive procedures (fillings, pulpotomy), and similarly, behavioural outcomes remained statistically non-significant throughout familiarisation, preventive, and restorative appointments. Non-significant findings suggest that PPA is not universally effective, and that for many children—especially older or temperamentally independent ones—the dentist–child interaction is a stronger predictor of cooperation than the mere presence of a parent.

Moreover, the type or invasiveness of the dental procedure did not significantly predict PPA effectiveness. Null or non-significant findings were reported for a wide range of treatments, from prophylaxis and examinations to pulpotomies and extractions [22,23,24,25].

This indicates that procedural intensity alone does not modulate the behavioural effect of parental presence; instead, emotional regulation, prior dental experiences, and attachment security are likely stronger determinants.

### 4.3. Negative or Counterproductive Effects

A smaller number of studies reported potentially counterproductive effects of parental presence. Cox et al. [21] observed more disruptive behaviour among younger children when a parent was present, and Al-Namankany et al. [29] also found higher anxiety levels in children treated with their parents in the operatory.

These significant results suggest that, for certain children—particularly those exposed to parental anxiety or excessive attention—PPA may increase emotional reactivity and hinder independent coping.

### 4.4. Behavioural, Physiological, and Methodological Variability

Physiological indicators of anxiety produced mixed and often non-significant findings. Shindova and Belcheva [23] found no statistically significant differences in heart rate between parental presence and absence, while Pani et al. [24] observed that physiological arousal (heart rate) was higher in children accompanied by parents (*p* < 0.05), although behavioural scores showed no significant difference.

This inconsistency—where physiological arousal diverges from overt behaviour—suggests a partial dissociation between emotional and behavioural responses, complicating the interpretation of PPA outcomes.

Bagavathy et al. [30] found a significant association between active parental involvement and reduced anxiety, whereas passive presence produced no measurable benefit. Although this study compared the quality rather than the existence of parental presence, its results highlight that the type and engagement level of parental participation can influence behavioural outcomes.

When the PPA approach was applied to manage uncooperative behaviour in children during dental procedures, no significant improvement in behaviour was observed Boka et al. [25].

### 4.5. Parenting Style as a Moderating Variable

Parenting style has emerged as a statistically and clinically relevant factor influencing the behavioural impact of parental presence. Gera et al. [35] observed significant differences (*p* < 0.05) in child behaviour depending on parental style: children from authoritative households—characterised by warmth and consistent limits—responded best to techniques involving active parental support, including parental presence; those from authoritarian homes benefited more from parental separation; and children of permissive parents responded better to distraction.

These findings suggest that the effectiveness of PPA depends not only on the child’s age or temperament but also on the quality of the parent–child interaction. In other words, parental presence is more likely to be beneficial when the parent’s behaviour models calmness and structure, rather than anxiety or overcontrol. Hence, the impact of PPA cannot be understood as a simple presence–absence dichotomy but as part of a broader socio-familial dynamic that shapes emotional regulation during dental treatment.

### 4.6. Cultural and Contextual Influences

Cultural factors may partly explain the heterogeneity of PPA outcomes. Studies conducted in countries where parenting norms tend to emphasise obedience and authority (e.g., Iran, India, Turkey) generally reported non-significant or less favourable behavioural effects of parental presence. Conversely, research from more autonomy-supportive contexts (e.g., Greece, the Netherlands) tended to show more positive or cooperative responses. This pattern is consistent with cross-cultural findings indicating that parental expectations about independence, emotional expression, and control shape the quality of parent–child interactions during dental treatment, and consequently the behavioural impact of PPA.

### 4.7. Emerging Trends and Clinical Implications

A chronological pattern is also evident: more recent studies (2020–2024) tend to report more positive or statistically significant outcomes of PPA, possibly reflecting shifting parental norms towards emotional support and protective engagement. Such cultural evolution may enhance children’s dependency on parental proximity and alter their stress response to separation.

Clinically, the evidence indicates that decisions about parental presence should be individualised rather than prescriptive. PPA appears statistically and clinically beneficial for children showing separation anxiety, limited emotional regulation, or previous negative dental experiences. Conversely, no benefit or even adverse effects may occur in children who are overstimulated by parental attention or capable of autonomous self-regulation.

Therefore, paediatric dentists are advised to conduct brief pre-treatment assessments considering age, temperament, cognitive maturity, and parental demeanour.

Adopting an evidence-informed, context-sensitive approach enables clinicians to select the most appropriate behavioural strategy—maximising cooperation and minimising anxiety—based on the child–parent–dentist triad.

### 4.8. Limitations

The evidence base is limited by methodological heterogeneity, including variations in behavioural scales, sample sizes, and clinical procedures, which complicate cross-study comparison. Several studies showed design weaknesses such as selection bias in RCTs [23,31], lack of blinding [21,22,23,24,25,26,28,31], and inadequate control of confounders in cohort and cross-sectional studies [19,20,29,35]. The inclusion of broad age ranges (e.g., 2–14 years) may have introduced developmental variability affecting behavioural responses to PPA. Moreover, the absence of longitudinal data prevents evaluation of whether behavioural effects persist over time.

The incorporation of studies examining related constructs—such as the use of parental presence versus parental separation to manage disruptive behaviour [25], active versus passive parental presence [28] or broader notions of parental involvement [30]—may have further increased heterogeneity due to inconsistent definitions. Nonetheless, these studies were retained for their conceptual relevance. Publication and language bias cannot be excluded, as the search was limited to English-language sources.

Finally, the marked heterogeneity across studies meant that a meta-analysis could not be conducted, preventing the estimation of pooled effect sizes or heterogeneity indices (e.g., I^2^).

### 4.9. Future Research Directions

In view of these limitations, future research should prioritise well-designed randomised controlled trials with standardised behavioural and anxiety outcome measures to allow meaningful comparison and potential meta-analysis. Studies should also explore moderating variables such as parenting style, cognitive development, and cultural context using robust multivariate models. Furthermore, longitudinal research is needed to determine whether the behavioural effects of parental presence persist over time and across different stages of child development. Finally, qualitative studies could provide deeper insight into the emotional dynamics within the parent–child–dentist triad, helping to refine personalised behaviour guidance strategies.

## 5. Conclusions

This systematic review did not find evidence of a significant global effect of PPA on children’s behaviour in the dental setting. Although several studies reported improvements directionally positive outcomes—particularly among younger children (3–6 years), those with initially negative or uncooperative behaviour, and children with higher cognitive ability—these subgroups were more likely to show improved cooperation or reduced distress in several RCTs and cohort studies, whereas older children and those with more severe baseline behavioural difficulties generally did not experience measurable benefit.

Findings concerning anxiety are similarly mixed: while some studies indicate potential reductions in physiological arousal or emotional distress when parents are present, others reveal no measurable difference or, in a few cases, less favourable outcomes.

Taken together, current evidence points towards the value of a selective and individualised use of PPA, adapted to the child’s developmental and emotional characteristics and the quality of the parent–child relationship, rather than its systematic application. Further research employing standardised behavioural measures, longitudinal designs, and culturally diverse samples is needed to clarify the specific contexts in which parental presence may optimise cooperation and emotional regulation in paediatric dental care.

## Figures and Tables

**Table 1:** Methodological quality assessment of cohort studies using the JBI checklist.

Study	Q1	Q2	Q3	Q4	Q5	Q6	Q7	Q8	Q9	Q10	Q11	Overall Quality
Kotsanos et al. [19]	Yes	Not applicable	Not applicable	No	No	No	Yes	Yes	No	No	Yes	Moderate–Low
Kotsanos et al. [20]	Unclear	Yes	Not applicable	Unclear	No	Yes	Yes	Yes	Unclear	Unclear	Yes	Moderate–Low
Al-Namankany et al. [29]	Yes	Yes	Yes	Yes	No	Yes	Yes	Yes	no	No	Yes	Moderate

**Table 2:** Methodological quality assessment of RCTs according to the JBI critical appraisal checklist.

Study	Q1	Q2	Q3	Q4	Q5	Q6	Q7	Q8	Q9	Q10	Q11	Q12	Q13	Overall Quality
Cox et al. [21]	Yes	Unclear	Yes	No	No	Yes	Unclear	Yes	Yes	Yes	Yes	Yes	Yes	Moderate
Afshar et al. [22]	Yes	Unclear	Yes	No	No	Yes	Unclear	Yes	Yes	Yes	Yes	Yes	Yes	Moderate
Shindova & Belcheva [23]	Unclear	No	Yes	No	No	No	Yes	Unclear	Yes	Yes	Yes	Yes	No	Low
Pani et al. [24]	Yes	Unclear	Yes	No	Unclear	Yes	Unclear	Yes	Yes	Yes	Yes	Yes	Yes	Moderate
Boka et al. [25]	Yes	Unclear	Yes	No	Unclear	Yes	Unclear	Yes	Yes	Yes	Yes	Yes	Yes	Moderate
Boka et al. [26]	Yes	Unclear	Yes	No	Unclear	Yes	Unclear	Yes	Yes	Yes	Yes	Yes	Yes	Moderate
Ahuja et al. [3]	Unclear	Unclear	Unclear	Unclear	Unclear	Unclear	Unclear	Yes	Yes	Not applicable	Unclear	Yes	Unclear	Uncertain
AlDhelai et al. [28]	Yes	Unclear	Yes	No	Unclear	Yes	Unclear	Yes	Yes	Yes	Yes	Yes	Yes	Moderate
Karaca & Şirinoğlu Capan [31]	Unclear	Unclear	Yes	No	Unclear	Yes	Unclear	Yes	Yes	Not applicable	Unclear	Yes	Yes	Moderate

**Table 3:** Methodological quality assessment of cross-sectional studies using the JBI checklist.

Study	Q1	Q2	Q3	Q4	Q5	Q6	Q7	Q8	Overall Quality
Acharya et al. [4]	Yes	Yes	Yes	Yes	No	No	Yes	Yes	Moderate
Shiraz et al. [27]	Yes	Yes	Yes	Yes	No	No	Yes	Yes	Moderate
Bagavathy et al. [30]	Yes	Yes	Yes	Yes	No	No	Yes	Yes	Moderate
Gera et al. [35]	Yes	Yes	Yes	Yes	No	No	Yes	Yes	Moderate

## Data Availability

The datasets generated and analysed during the current study are available from the corresponding author upon reasonable request.

## Supplementary Materials

The following supporting information can be downloaded at: https://www.mdpi.com/article/10.3390/dj14010033/s1. Table S1: PRISMA 2020 Checklist.

## Abbreviations

The following abbreviations are used in this manuscript:

PPA	Parental Presence/Absence
PAP	Parental Active Presence
PPP	Parental Passive Presence
PPAT	Parental Presence/Absence Technique
RCT	Randomized Controlled Trial
M/F	Male/Female
P/A	Parent Present/Absent
NI	Not Informed
HR	Heart Rate
LA	Local Anaesthesia
MDAS	Modified Dental Anxiety Scale
CFSS-DS	Children’s Fear Survey Schedule—Dental Subscale
VAS	Venham Anxiety Scale
Frankl	Frankl Behaviour Rating Scale
WBFPS	Wong–Baker Faces Pain Rating Scale
FIS	Facial Image Scale
ACDAS	Abeer Children’s Dental Anxiety Scale
IQ	Intelligence Quotient
JBI	Joanna Briggs Institute
PRISMA	Preferred Reporting Items for Systematic Reviews and Meta-Analyses

## Appendix A

**Table A1:** Key findings on parental presence vs. absence and child behaviour during dental treatment.

Author	Year	Country	Study Design	Setting	N (M/F) (P/A)	Age (Years)	Procedure/Visit Length/Parent Position	Behaviour/Anxiety/Fear Scales/Measures	First Visit	Main Findings	Parent Presence Effect
**Kotsanos et al. [19]**	2005	Greece	Cohort study	Paediatric dental practice	85 (NI)	2.7–8.8	Initial and follow-up visits	Frankl Behaviour Rating Scale	Yes	94% behavioural improvement (*p* < 0.001). Effective for initially uncooperative children using the PPA technique; clear behavioural improvement observed.	Better
**Kotsanos et al. [20]**	2009	Greece	Cohort study	Paediatric dental practice	440 (NI)	3–10	Initial and follow-up visits	Frankl Behaviour Rating Scale	Yes	Improved behaviour in mild and moderate cases but not in severe ones. Early application of PPA was partially effective and more beneficial in less severe behavioural problems.	Better (mild/moderate)
**Cox et al. [21]**	2011	Netherlands	RCT	Paediatric dental clinic	90 (45M/45F) (47P/43A)	4–12	Habituation and treatment sessions	Venham behaviour scale, CFSS-DS, Wong-Baker Faces	No (3 visits)	Children aged 4–5 showed more negative behaviour with parents (*p* = 0.0049), whereas older children showed no difference. Parental presence may increase reactivity in anxious younger children.	4–5: Worse; 6–12: No difference
**Afshar et al. [22]**	2011	Iran	RCT	Dental school, paediatric dept.	67 (33M/34F) (32P/35A)	5	1st: prophylaxis/fluoride; 2nd: pulpotomy/fillings (30 min)	Venham Anxiety Scale, Frankl	Yes	No significant difference (*p* > 0.05) in children’s behaviour or anxiety between parental presence and absence. PPA did not influence outcomes.	No difference
**Shindova & Belcheva [23]**	2013	Bulgaria	RCT	Faculty of Dental Medicine,	48 (NI) (24P/24A)	6–12	Clinical oral examination; parent present or absent	Heart rate, oxygen saturation, self-report Faces Scale	Yes	Heart rate increased slightly but anxiety decreased, with no statistically significant differences between groups (*p* > 0.05). Parental presence had no measurable effect on anxiety in 6–12-year-olds.	No difference
**Pani et al. [24]**	2016	Saudi Arabia	RCT	Dental school, paediatric dept.	122 (62M/60F) (47Fathers/42Mothers/33A)	6–8	First restorative dental visit	Venham Anxiety Scale, Venham Fear Scale, Heart rate	Yes	Lower physiological anxiety when parents were absent (*p* < 0.05), but behavioural scores remained unchanged (*p* > 0.05). Parental presence reduced physiological anxiety but did not modify cooperative behaviour.	Anxiety: Worse; Behaviour: No difference.
**Boka et al. [25]**	2016	Greece	RCT	Postgraduate dental school	100 (49M/51F) (50P/50A)	4–12	Familiarization + ≥2 sessions	MDAS, CFSS-DS, modified Venham, Wong-Baker	No	No significant differences (*p* > 0.4). Parental presence did not affect behaviour, although children reported slightly higher comfort levels.	No difference
**Boka et al. [26]**	2017	Greece	RCT	Postgraduate dental school	61 (27M/34F) (30P/31A)	3–8	Prevention, restoration, extraction. PPA	Frankl Behaviour Rating Scale	No	No significant behavioural differences were found; a slight improvement was observed in children with previous negative dental experiences. PPA was not superior to other non-pharmacological techniques.	No difference
**Ahuja et al. [3]**	2018	India	RCT	Hospital dental clinic	30 (19M/11F)	4–7	Two simple restorative visits (parent present 1st, absent 2nd)	Frankl Behaviour Rating Scale	Yes	Cooperation appeared to improve mainly due to individual or family factors; preschoolers were initially more negative. Parental presence did not enhance behaviour and might not always be beneficial.	No significant difference; younger children slightly less cooperative with parents
**Acharya et al. [4]**	2019	India	Cross-sectional	Dental school, paediatric dept.	60 (NI) (30P/30A)	3–12	Routine dental procedures	Frankl Behaviour Rating Scale	Yes	Younger children (3–5 years) behaved better with parents present, while older children showed no difference. PPA benefited only the younger group.	3–5 years: Better; 6–12 years: No difference
**Shiraz et al. [27]**	2020	Pakistan	Cross-sectional	College of Dentistry,	61 (NI)	2–14	PPA applied during first dental visit	Frankl Behaviour Rating Scale	Yes	Behavioural cooperation improved significantly in the 4–6 year group (*p* = 0.035). PPA enhanced cooperation in this age range.	Better (4–6 years)
**AlDhelai et al. [28]**	2021	Egypt/Saudi Arabia	RCT	University paediatric dental clinic	150 (75 M/75 F) (75 PAP/75 PPP)	3–6	1st visit: screening & IQ testing (Stanford–Binet IV); 2nd visit: preventive procedures (prophylaxis, fissure sealant, topical fluoride). PAP. PPP	Frankl Behaviour Rating Scale FBRS, FIS, Stanford–Binet IV (IQ stratification: low/average/high)	No (preventive second visit)	Positive behaviour was observed in 74.7% of children in the active parental presence (PAP) group versus 46.7% in passive presence (PPP) (*p* < 0.0001). Active parental participation significantly improved cooperation, especially in children with higher IQ.	Better (significant)
**Al-Namankany [29]**	2023	Saudi Arabia	Cohort study	Paediatric dental clinic	84 (43M/41F) (42P/42A)	6–9	Composite restoration under local anaesthesia	Abeer Children’s Dental Anxiety Scale (ACDAS)	No (2 visits)	Children showed higher anxiety levels with parents present (*p* < 0.001). Parental absence improved behaviour, and the PPAT technique was most effective for anxious children.	Absence: Better
**Bagavathy et al. [30]**	2024	India/Saudi Arabia (collaborative)	Cross-sectional study	Paediatric tertiary care centre	100 (NI)	4–10	Routine dental treatments; categorised as high, moderate, or low parental interaction	Validated behaviour rating scale (cooperation, anxiety, disruptive behaviour)	NI	Children with high parental involvement demonstrated significantly higher compliance (mean 8.5 vs. 5.8; *p* < 0.001). Active parental engagement improved cooperation and reduced anxiety.	Better (significant)
**Karaca & Şirinoğlu Capan [31]**	2024	Turkey	RCT	University paediatric dental clinic	194 (92 M/102 F) (102 P/92 A)	5–8 (mean 6.26 ± 1.15)	Restorative treatment in primary first molars (~20 min); Group I parent present vs. Group II absent	Modified Dental Anxiety Scale (MDAS), Wong-Baker Faces Pain Rating Scale (WBFPS), Heart Rate	Yes	No statistically significant differences were found in anxiety or heart rate (*p* > 0.05). Behaviour was slightly better with parents present but not statistically significant.	No difference (non-significant)
**Gera et al. [35]**	2025	India	Cross-sectional study	Dental school, paediatric dept.	120 (NI)	4–8	Restorative treatment; behaviour management techniques tested sequentially (tell-show-do, distraction, parental presence, parent separation, voice control)	Frankl Behaviour Rating Scale, Facial Image Scale	Yes	Children of authoritative parents responded best to tell–show–do; those from authoritarian homes benefited more from separation, and those from permissive households from distraction. Parental presence was effective only in a minority of authoritarian families, indicating that parenting style modulates the PPA effect.	Mixed: Effective only in specific contexts (e.g., supportive/authoritative parents); less effective for authoritarian styles
